# Patterns of DNMT1 Promoter Methylation in Patients with Acute Lymphoblastic Leukemia 

**Published:** 2017-07-01

**Authors:** Tirdad Rahmani, Mehdi Azad, Bahram Chahardouli, Hajar Nasiri, Mousa Vatanmakanian, Saeid Kaviani

**Affiliations:** 1 Department of Hematology, School of Medical Sciences, Tarbiat Modares University, Tehran, Iran; 2 Department of Medical Laboratory Sciences, Faculty of Allied Medicine, Qazvin University of Medical Sciences, Qazvin, Iran; 3 Hematology-Oncology and Stem Cell Transplantation Research Center, Tehran University of Medical Sciences, Tehran, Iran; 4 Children’s Medical Center, Tehran University of Medical Sciences, Tehran, Iran; 5 Department of Hematology, School of Allied Medical Sciences, Tehran University of Medical Sciences, Tehran, Iran

**Keywords:** Acute lymphocytic leukemia, Epigenetic, Methylation, DNA methyltransferase

## Abstract

**Background: **Acute lymphoblastic leukemia (ALL) is a clonal malignant disorder characterized by an uncontrolled proliferation of immature T or B lymphocytes. Extensive studies have shown that the epigenetic changes, especially modified DNA methylation patterns in the regulatory regions through the DNA methyltransferase (DNMTs), play an important role in the development of genetic disorders and abnormal growth and maturation capacity of leukemic stem cells (LSCs).The aim of this study was to evaluate the changes in DNMT1 promoter methylation and its expression pattern in patients with ALL.

**Materials and Methods: **In this experimental study, methylation specific PCR (MSP) was used to assess the methylation status of DNMT1 promoter regions in samples collected from ALL patients (n=45) and healthy control subjects. According to this method, un-methylated cytosine nucleotides are converted to uracil by sodium bisulfite and the proliferation of methylated and un-methylated regions are performed using specific primers for target sequences.

**Results: **None of the patients with B and T-ALL showed methylated promoter regions of the DNMT1 gene, while the methylation pattern of both pre-B ALL patients and the control group showed a relative promoter methylation.

**Conclusion: **Analysis of promoter methylation patterns in various subgroups of ALL has revealed the importance of DNMT1 in the regulation of gene expression. Likewise, extensive data have also highlighted the methylation-based mechanisms exerted by DNAM1 as one of the main participants regulating gene expression in B-ALL and T-ALL patients. Investigation of the overall DNA methylation pattern offers significant improvements in the prediction of disease prognosis and treatment response.

## Introduction

 Acute lymphoblastic leukemia (ALL) is a clonal hematopoietic stem cell disorder, caused by several geneticchanges that increased the production of immature lymphoid cells. It is associated with a wide spectrum of molecular and clinical heterogeneities, as the different types of mutations may increase the risk of developing the disease^   1 ^. Early events through the evolutionary origin of abnormal lymphocytes begin by escaping the normal growth regulation mechanisms and gene defects involved in lymphoid proliferation and differentiation as well as epigenetic modifications. Therefore, leukemic stem cells (LSCs) apply all the possible ways to prevent the cell death and maturation toward the mature lymphocytes^   2 ^**.**

Although many molecular or genetic studies have been conducted for better understanding of pathogenesis and biology of lymphoid malignancies, the detailed analysis of DNA methylation patterns may be helpful for an explanation of the underlying regulatory mechanisms of the disease^   3 ^. DNA methylation in eukaryotic cells, one of the significant mechanisms of the epigenetic modifications, occurs with the addition of a methyl group to the 5 positions of the cytosine ring in CpG islands. The variability of DNA methylation patterns is achieved due to the multiplicity and functional diversity of DNA methyl transferase enzymes (DNMTs), which regulate the methylation of cell cycle and DNA repair gene promoters in accordance with other cell physiological processes^   4 ^. Regarding the essential role of different DNMT isoforms, especially DNMT1 in regulation of hematopoietic stem cell (HSC) growth, differentiation and survival, it is not surprising that the aberrant methylation of the enzyme result in leukemogenesis^   5 ^.

Similar studies using inhibition of DNMT1 by allelic exclusion or pharmaceutical contribution have concluded that the abnormalities of these enzymes have a directly negative impact on the self-renewal and differentiation of LSCs but not on normal HSCs^   6 ^.

In recent years, great efforts have been made aiming to identify the epigenome map and the DNA methylation patterns in patients with ALL, which have suggested the aberrant methylation of several genes controlling the cell cycle and signal transduction between different subtypes of the disease^   7 ^. Although it has been recognized that the abnormality remains relatively constant during the remission phase, the likelihood of recurrence increases following the treatment^8^^,^^9^.

Despite the existence of classification systems for the identification of high-risk ALL patients, the differential diagnosis between the recurrent events and the stable cases appears to be challenging.

Since the individuals with epigenetic modifications may be predisposed to leukemogenesis^   10 ^, the question now arises whether the promoter methylation level of the target gene can be used to help determine the stage of the disease. We have studied the promoter methylation level of DNMT1 gene among the various subgroups of ALL, aiming to clarify if the methylation assay of this gene can be used to predict the outcomes of the patients with poor prognosis. Our data may suggest further studies to evaluate novel therapeutic strategies such as DNMT modifying agents to control the ALL progression in early-stage patients.

## MATERIALS AND METHODS

**Sample collection**

Peripheral blood samples were obtained from 45 newly diagnosed ALL patients who were referred to Hematology-Oncology and Stem Cell Transplantation Research Center, Shariati Hospital in Tehran as well as Quds and Razi Hospitals in Qazvin, Iran. Peripheral blood cells from 12 healthy volunteers were used as controls (6 adults and 6 children).Written consent forms were signed by the participants or their parents. A flow cytometry-based assay was used to subdivide ALL patients according to the guidelines published by the World Health Organization (WHO). Nearly, 68.9% of patients belonged to pre-B ALL category, while 24.4% and 6.7% of patients belonged to T-ALL and B-ALL, category, respectively.

**DNA extraction and preparation**

DNA extraction was carried out with a column-based centrifugation procedure according to the manufacturer’s protocol of GeneAll Kit (GeneAll Biotechnology, Seoul, Korea). The DNA pool was bisulfite treated using the EpiTect Bisulfite Kit (Qiagen) and prepared for the methylation-specific PCR (MSP) assay. The unmethylated cytosine bases are converted to uracil, while 5-methylcytosine bases remain intact during the bisulfite treatment. Also, to reduce the loss of DNA after modification, molecular biology-grade glycogen was added as a co-precipitant to offset the decline in DNA concentration.

**DNA quality control**

The quality of DNA extracted from the blood samples and also during the sodium bisulfite treatment, as well as glycogen clean-up process were also assessed by the UV spectrophotometry (NanoDrop) and agarose gel electrophoresis.

**Methylation-specific PCR (MSP)**

MSP is a simple and inexpensive method for the evaluation of DNA methylation patterns. In this method, unmethylated and methylated regions of DNA are amplified following the sodium bisulfite treatment. MSP was performed for each studied sample, using two pairs of primers specific for the methylated (M) and unmethylated (U) sequences (Table 1). The primer sequences had been designed in previous studies^   11 ^. Amplification of the template using M and U primers indicates the methylated and unmethylated CpG islands, respectively, while the amplification of both M and U primers indicates the partial methylation of target sequences. The SssImethylase enzyme (New England Biolabs) and the untreated genomic DNA was used as positive and negative controls through this approach. The PCR products were then confirmed by 1.5% agarose gel electrophoresis.

**Table 1:** DNMT1 primer sequences for MSP

**Gene**		**Size**	**Cycles**	**Temp**	**Form**	**5` to 3` sequences**
**DNMT1**	280		35	54 58.2	MF MR	AGTAAATTGTGGAGTTTGGATGAG TTTA AACACAAACACCCCAACTTTTCACA CG
	288		35	54 56.7	UF UR	AGTAAATTGTGGAGTTTGGATGAG TTTA AACACAAACACCCCAACTTTTCACA CA

The obtained results statistically analyzed using SPSS version 20. A p-value of <0.05 was considered significant.

## Results

 The promoter methylation status was analyzed, using MSP method in 45 patients; of whom 26 (57.7%) were male and 19 (42.2%) were female.

**Figure 1:**
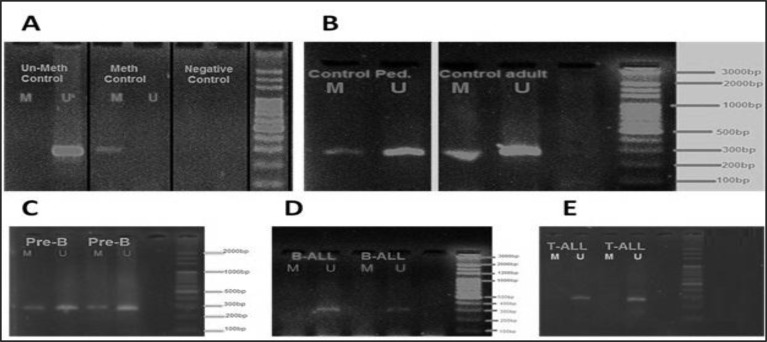
DNMT1 promoter methylation pattern in patients with ALL. **A)** Is indicating the methylated and un-methylated controls as well as a negative control which show the valid results. **B)** Indicates the MSP results of DNMT1 methylation status in healthy children and adults, which suggest a partial methylation pattern for DNMT1 promoter. **C)** MSP results of DNMT1 in patients with pre-B ALL observing as a partial methylation pattern resembling the healthy controls. **D)** Is indicating an un-methylated pattern for DNMT1 promoter in B-ALL patients. **E)** MSP results of DNMT1 in patients with T-ALL showing a clear un-methylated pattern of DNMT1 promoter for these patients. * M and U represent the reactions in which the Methylated and Un-methylated forms of primers were used, respectively.

The mean age of the patients was 26.6 years (range 4-59y). As shown in Figure 1, methylated and unmethylated forms of DNMT1 Gene product are represented as 280 bp and 288 bp in length.

The methylated and unmethylated forms of DNMT1 gene in three subgroups of T-ALL, B-ALL and pre-B ALL patients are shown in Figure 1. Controls for methylated and unmethylated sequences were used to ensure the validation of the results (Figure 1A). A sodium bisulfite untreated genomic DNA was used as the negative control sample (Figure 1A). Spots with the M and U letters are related to the reactions in which the primers are specific for the methylation and unmethylation status of the target sequences, respectively.

Figure 1B shows the results of the MSP products for the methylated and unmethylated forms of target sequences among the healthy individuals as control groups. As the DNA methylation patterns may be diverse in different ages and between two sexes, the control group was selected from an age- and gender-matched population.

**DNMT1 is partially methylated in Pre B-ALL patients and healthy individuals**

The results of MSP experiments indicated a clear sharp band in both methylated and unmethylated forms, partial methylated status of promoter regions and relative expression of the DNMT1 gene among the healthy subjects. We have also observed clear MSP bands in both methylated and unmethylated forms in Pre B-ALL patients (Figure 1C), indicating a partial methylation status and a relative expression of this gene.

**DNMT1 is in non-methylated form in B- and T-ALL patients**

Despite having a partial methylation form in preB-ALL cases and healthy individuals, DNMT1 seems to be in an unmethylated form in B-ALL and T-ALL patients. As shown in Figure 1D and E, the MSP products were exclusively observed in the unmethylated forms of the promoter region, indicating an absence of promoter DNA methylation of DNMT1 in these patients. The unmethylated status observed in the B and T-ALL patients may suggest a highly expressed DNMT1 in these patients which requires further molecular analysis to reveal the expression levels in parallel. Pediatric controls and adult controls are used to indicate the healthy children and adults, respectively. Pre-B/Ped is related to the children with pre-B ALL. No difference was observed concerning the methylation pattern among different age groups (p=0.122) and between male and female subjects (p=0.181).

## Discussion

 DNA methylation plays an important role in the expression of several genes that are involved in cell growth and differentiation, as well as genomic integrity since epigenetic changes of this type hugely support the production of mature lymphocytes with efficient antigen receptors through the lineage commitment of lymphoid precursor cells^12^. This finding is also consistent with previous studies of the patterns of B-cell activation in the germinal center (GC) of lymphoid follicles since the significant expansion of lymphocyte subset associated genes are hypomethylated in such regions^   13 ^. GC B cells undergo selection and affinity maturation process with high proliferation rates. It may therefore be expected that the dysfunction in lymphocyte evolutionary gene expression may contribute to leukemogenesis.

Analysis of the methylation status of DNMTs as a novel approach to identify the high-risk patients with B-cell lymphoma has revealed increased activity of DNMTs among patients diagnosed with this disease^14^.

T cells appear to follow this program since the decreased expression of DNMTs and therefore the occurrence of DNA hypomethylation in such cells may lead to the increased expression of cytokines such as IFN-γ and IL-2^   15 ^. In addition to the regulatory role of IL-2 on T cell growth and differentiation, it is widely considered as one of the key cytokines involved in T cell activation through its influence on the proper selection and inhibition of leukemic cell growth^16^^,^^17^.

Increased expression of DNMTs and consequently aberrant methylation of cell cycle regulatory genes have been reported in a number of hematologic malignancies. Activation of these enzymes in LSCs results in the hypermethylation of cyclin-dependent kinase inhibitor p15^INK4B^ in patients with acute myeloid leukemia (AML) and acute phase of chronic myeloid leukemia (CML)^   18 ^. These results are partially in parallel with our study since the reduced DNMT1 promoter methylation is completely evident in both T-ALL and B-ALL patients. Considering the prerequisite role of cell cycle dysregulation in LSC formation, aberrant epigenetic network, especially abnormal DNMT1 methylation, may lead to the defects in ALL.

On the other hand, the lack of DNMT1 expression is an underlying cause of several problems associated with HSCs because the epigenetic modifications, particularly DNA methylation, has a fundamental role in self-renewal and differentiation of HSCs toward various cellular components^   19 ^. In fact, the lack of DNMT1 enzyme expression leads to loss of cell cycle regulation and lineage commitment during the successive cell divisions in this background^   20 ^. This defect is more apparent in lymphoid cell lineages, especially T cells compared to myeloid progenitor cells, and acts as a barrier against their differentiation process. Regulation of HSC self-renewal and differentiation is made through the direct cell interactions with bone marrow stromal cells and the HSCs deployment in bone marrow niche has a big impact on their fate determination^   21 ^^-^^23^.

Our study revealed an unmethylated pattern of DNNT1 promoter in B-ALL and T-ALL patients that may be linked to the poor prognosis in such cases. However, it is remarkable that pre-B ALL cases have a different methylation pattern from the B-ALL and T-ALL patients. Investigations focused on ETV6-RUNX1-positive pre-B ALL children showed the aberrant methylation profile of tumor suppressor genes involved in ALL^24^^,^^25^. The result of our study raises the possibility whether other DNA methyltransferases such as DNMT1 are responsible for controlling LSC gene expression pathways, or pre-B ALL cells are also used a different way to influence the components of cell cycle-controlling network. It appears that further studies regarding the quantitative assessment of DNMT1 promoter methylation among the different subtypes of ALL, and the comparison of their methylation pattern with expression profiles of genes involved in disease recurrence would also be helpful for the accurate prediction of disease progression and response to treatment.

## CONCLUSION

 Taken together, in this study we have reported an unmethylated status in DNMT1 gene promoter region of B- and T-ALL patients. As a result, DNAMT1, as one of the central elements of epigenetic machine which controls the gene regulation during leukemogenesis, was shown itself to be controlled via the methylation mechanism. Investigation of the overall DNA methylation pattern offers significant improvements in the prediction of disease prognosis and treatment response.
